# Mortality and Its Predictors among HIV Infected Patients Taking Antiretroviral Treatment in Ethiopia: A Systematic Review

**DOI:** 10.1155/2017/5415298

**Published:** 2017-10-30

**Authors:** Mohammed Biset Ayalew

**Affiliations:** Department of Clinical Pharmacy, School of Pharmacy, College of Medicine and Health Sciences, University of Gondar, Gondar, Ethiopia

## Abstract

**Background:**

Even though the benefit of antiretroviral therapy (ART) is well established, there is a regional variation in the extent of its benefit. The aim of this review is to highlight mortality and its predictors in Ethiopian adult HIV patients who were on ART.

**Methods:**

Relevant articles were searched on PubMed and Google Scholar databases. The search terms used in different combinations were predictor/determinant/factors, mortality/death/survival, HIV, ART/HAART, and Ethiopia.

**Result:**

5–40.8% of the patients died during the follow-up period. More than half (50–68.8%) of the deaths occurred within 6 months of initiating ART. Advanced stage disease (stage III and stage IV), nonworking functional status (bedridden and ambulatory), low baseline CD4 count, low baseline hemoglobin level, TB coinfection, lower baseline weight, and poor treatment adherence were commonly identified as predictors of death in HIV patients.

**Conclusion:**

5–40.8% of HIV patients in Ethiopia die in 2–5 years of initiating antiretroviral treatment. Most of the deaths in HIV patients occur early in the course of treatment. Special emphasis should be given for patients with advanced stage disease, nonworking functional status, low baseline CD4 count, low baseline hemoglobin level, TB coinfection, lower baseline weight, and poor treatment adherence.

## 1. Introduction

HIV/AIDS is among the primary public health challenges that have affected the world's social, economic, and political system in the recent past. During the last three decades, millions of people died due to HIV infection. In the year 2012, more than 1.6 million AIDS deaths were recorded [1]. The burden of the disease is very high in the sub-Saharan African region. Even though only 12.5% of the world's population is living in this region, about 68% of the world's HIV infected population is found there [2]. According to a 2013 report, the sub-Saharan African region covers 74% of HIV-related deaths [3]. Ethiopia, as one of the sub-Saharan region countries, has one of the largest populations of HIV infected people in the world [4]. There are about 1.2 million HIV infected people in Ethiopia. An estimated prevalence of HIV/AIDS in the adult Ethiopian population is 2.4% with the incidence rate of 0.29% [5].

The introduction of antiretroviral therapy (ART) significantly improved the survival of HIV patients [6] and changed HIV infection from a fatal illness to a manageable chronic disease [7]. ART suppresses viral replication, restores immune function, reduces HIV associated morbidity and mortality, increases quality of life, and prolongs survival [1]. Because of these benefits of ART, there is a high effort globally to increase its coverage. As a result, access to ART was improved even in resource constrained countries. Ethiopia has been also struggling on the scale-up of ART in order to increase its access to all people with HIV/AIDS. ART was introduced in Ethiopia as a subsidized, fee-based scheme in 2003 and two years later (in 2005) it was made freely available. Furthermore, in 2006, the service was decentralized to health centers which facilitated the rapid expansion of the ART program throughout the country [8].

Even though the benefit of ART for people living with HIV/AIDS in terms of improving quality of life and reducing morbidity and mortality is well established, there is a regional variation in the extent of its benefit. A significant number of mortalities in HIV patients were also recorded within a few years of starting ART. This early mortality is higher in resource constrained settings. A high rate of early mortality was reported from a number of sub-Saharan African ART programs [9–11]. Different factors were attributed in various studies to the occurrence of death in patients who already started their ART. There are various studies conducted in Ethiopia to determine the mortality rate and its determinants in HIV patients who started ART. Summarizing the findings from these studies will show the overall picture of mortality in HIV patients who are on ART in Ethiopia and the main contributing factors to death. Better knowledge of factors/predictors of mortality is essential as it allows closer follow-up and facilitates targeted interventions in patients who have a higher risk; as a result, mortality will be reduced [11–13]. So, the aim of this study is to determine the overall mortality rate in adult HIV patients who were already on ART and summarize the main predictors of mortality in these patient groups in Ethiopia.

## 2. Methods

### 2.1. Search Strategy

Studies conducted on mortality and its predictors among HIV patients who had started ART were searched on PubMed and Google Scholar databases. Additional articles were also obtained from the reference lists of retrieved articles and manual Google search. Retrospective cohort studies conducted on adult HIV patients in Ethiopia and written in English language were included. No restriction was applied on the year of publication. The following search terms were used in different combinations: mortality/death/survival, HIV, ART/HAART, predictor/determinant/factors, and Ethiopia.

### 2.2. Article Selection

Studies were included in this review if they assess mortality and factors associated with it among HIV infected patients taking antiretroviral treatment in Ethiopia. Studies which were conducted on pediatric populations and those studies which include HIV patients who did not start ART were excluded.

### 2.3. Assessment of Methodological Quality

Included studies were critically appraised by using the “STROBE Checklist” [14]. The checklist has 22 items which state the standards to be included in the cohort study. Values of 0, 1, and 2 were given for each study against each item: 0 means the study did not satisfy the criteria mentioned by that particular item, 1 means it partially fulfilled the criteria, and 2 means the study completely addresses the issue mentioned by the item. So, a single study was scored out of the maximum total value of 44 and expressed in terms of percentage. An article with a total score of 90% and more was considered to be of high quality, 75–89% indicated medium quality, and below 75% was considered as low quality.

### 2.4. Data Abstraction

Relevant information was obtained from the 17 studies by using a data extraction form. Author, year of publication, study area, study subjects, sample size, study design, median follow-up period, mortality incidence density/100 person-years (PY), mortality during the full follow-up period and at 3, 6, and 12 months, and factors affecting mortality with respective odds ratio (OR) were recorded in data abstraction format.

## 3. Result

### 3.1. Literature Search Results

**Figure 1:**
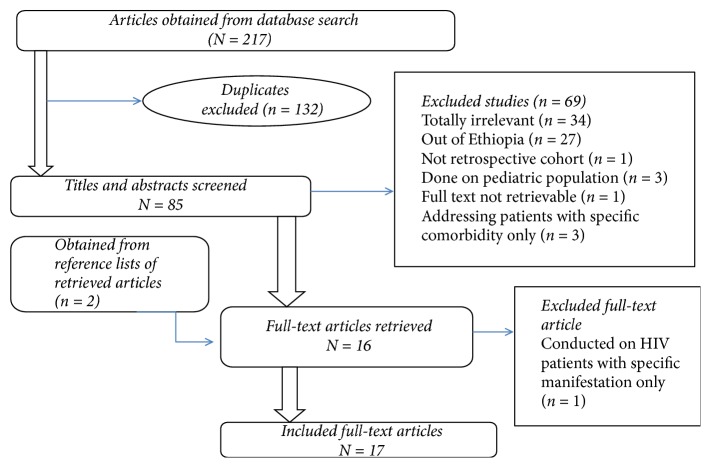
Article selection process.

A total of 217 articles were obtained from database (PubMed and Google Scholar) search, out of which 132 were duplicates. After screening the titles and abstracts of 85 studies, 69 were excluded. Two additional articles were obtained from reference lists of retrieved articles and one article was found to be of no importance after its full text was assessed. Finally, 17 articles were selected to be included in this review. The details of the article selection process are indicated in Figure 1.

### 3.2. Methodological Quality of Included Studies

After evaluation of each study against STROBE Checklist [14], fourteen studies were found to be of high quality and the remaining 3 were of medium quality. There was no significant difference in the mortality rate between the high and medium quality studies.

### 3.3. Study Characteristics

All of the studies included in this review were retrospective cohort studies conducted on adult HIV patients. A total of 19321 (range = 272–4210) study participants were included in the 17 studies reviewed. The detailed description of individual study characteristics is shown in Table 1.

### 3.4. Mortality

The included studies followed up patients for a median of 25–60 months with an average of 38.8 months of follow-up. As indicated in Table 2, 5%–40.8% of the patients died during the follow-up period. Most of the studies (82%) reported that 5–15% of the patients died within the follow-up period. The highest death rate recorded was 40.8% from Debremarkos General Hospital, while the minimum was 5% from South Gondar Zone. All of the studies which reported death rates at different points of follow-up indicated that more than half of the deaths occurred within 6 months of initiating ART. The mortality incidence density ranged from 0.2 to 10.74 per 100 person-years. Majority of the studies reported an incidence density of 1.89–5.3 per 100 person-years.

### 3.5. Predictors of Mortality

Among the demographic and clinical characteristics mentioned as a predictor for death in the reviewed studies, the most frequently mentioned were advanced stage disease (stage III and stage IV), nonworking functional status (bedridden and ambulatory), low CD4 count, low hemoglobin level, TB coinfection, poor adherence to ART, older age, lower weight, and lower baseline BMI. A detailed description of factors that predict mortality is indicated in Table 3.

Patients who started ART after they developed advanced stage disease are 1.4–11.2 times more likely to die than patients who started ART while they are in stage I or II. Patients who are bedridden and ambulatory are 2.4–6.9 and 1.4–4.2 times more likely to die than those who have a working functional status, respectively. Some studies express the effect of CD4 count as a continuous variable and demonstrated that the risk of death decreases by 1%–22% as the CD4 at initiation of ART rises by one unit. Other studies reported that CD4 < 50 cells/microliter at ART initiation increases the risk of death by 1.8–4.5 times. Lower hemoglobin level has 1.9–5.5 times increased risk of death. TB-HIV coinfection increases the risk of mortality 1.3–4.5-fold.

## 4. Discussion

Most of the studies (82%) included in the current review reported that 5–15% of the patients died within the respective follow-up periods. These rates of mortality were higher than what was reported in Uganda (4.5%) [15] and lower than the finding in Tanzania (29.7%) [13], Cameron (23%) [12], Berhampur (21.3%) [16], and Korea (20.8) [17]. The mortality incidence densities reported in the reviewed articles ranged from 0.2 to 10.74 per 100 person-years. Majority of the studies reported an incidence density of 1.89–5.3 per 100 person-years. This is in close agreement with the result of the study conducted in Ruanda which reported 3.7 per 100 person-years [18]. The mortality incidence density reported in this study is lower than the report from South Africa (12/100 PY and 17/100 PY) and Cameron (21.2/100 PY) [19]. The lower rate found in the current reviewed articles may be because of the absence of active search for patients who were lost to follow-up, and real case scenarios (only confirmed dead cases) were used as events in most of the studies reviewed.

All of the studies which reported death rates at different points of follow-up indicated that more than half (50%–68.8%) of the deaths occurred within 6 months of initiating ART. This indicated that most of the deaths in HIV patients occur early in the course of treatment. The reports that compare early mortality in HIV patients of low income and high income countries indicated that patients starting ART in resource constrained settings have increased mortality rates in the first months of therapy compared to those in developed countries [13, 20–22]. Several other studies in developing countries also demonstrated that mortality in the first few months of initiating ART was high [11, 23, 24]. The high early mortality rate observed in this review may be due to the fact that most of the patients included in most of the studies reviewed had either advanced stage disease or low CD4 cell count or both at initiation of their antiretroviral treatment so that they have higher probability of death due to serious opportunistic infections.

Many factors were found as a determinant of death. Among these, advanced stage disease, nonworking (bedridden and ambulatory) functional status, lower baseline CD4 count, lower baseline weight, lower baseline hemoglobin, TB coinfection, and poor adherence were frequently mentioned.

WHO clinical stage of the disease is the most important predictor of mortality reported by many of the studies included in this review. Many other studies conducted outside Ethiopia also reported the same result [11, 12, 25–28]. The higher mortality recorded in patients with advanced stage disease indicated that starting ART treatment earlier before the advancement of the disease is beneficial. But this needs an effort to early diagnose HIV infection by improving the counseling and testing practices. According to the result of this review, patients with bedridden and ambulatory baseline functional status were more likely to die than patients with working functional status. In this regard, many studies documented supporting evidence that poor performance status at the initiation of ART was a predictor of death during ART care [28–30].

Furthermore, the risk of death is higher for patients with low baseline CD4 count. The CD4 count is a reflection of the patients' immune status, so when it becomes low, the risk of developing opportunistic infections will increase, which may finally lead to death. Low CD4 count at initiation of ART was mentioned as the main predictor of death in HIV patients in various studies [11, 12, 31, 32]. Lower baseline hemoglobin level is also another very important predictor of mortality in HIV patients, which is reported by eight of the reviewed studies. Many other studies also support this finding [13, 25–27, 31, 33]. The possible explanation for increased mortality with low hemoglobin count may be due to the fact that the incidence of anemia increases with progression of HIV disease and most of the patients with low hemoglobin count had advanced stage disease which is a strong predictor of mortality in HIV patients.

Presence of TB coinfection is another important risk factor for death in HIV patients. According to Suchindran et al., the risk of death in TB-HIV coinfected individuals is double as compared to HIV infected individuals without TB [34]. Other studies also report that the presence of TB coinfection is significantly associated with higher odds of mortality among HIV patients taking ART [28, 35, 36]. This may be because TB by itself is a deadly disease worldwide, and the virulence of the organism is high in patients that have a suppressed immune system and are able to establish infection easily [37]. Low baseline weight and BMI of less than 18.5 was also found to be a predictor of mortality in many of the studies reviewed. This is in line with the result of studies conducted outside Ethiopia [11, 23, 38]. BMI is an indicator of patients' nutritional status. Patients with BMI less than 18.5 and lower weight are mostly malnourished and unable to cope with the disease and will have a high chance of death due to opportunistic infections.

Medication adherence is very important to get the full benefit of antiretroviral drugs. Nonadherence to ART will result in treatment failure by increasing the chance of mutation that could lead to a drug resistant virus and finally death. Even though a self-reported adherence assessment method was used in all of the reviewed studies, which is not as such reliable to measure adherence, some of the studies revealed that poor adherence is significantly associated with mortality. Those who did not have proper adherence to their ART medication were 2.2–27.8 times at greater risk of death than those who adhered to their medication. This was also reported in different studies conducted in various parts of the world [16, 31].

Age of patients was found to affect survival in HIV patients who are on ART. Four of the studies included in this review found significant association between age and death due to HIV after starting ART. Other studies also confirmed that most of the patients of older age were more likely to die [15, 39–41]. This may be due to the fact that as the patient gets older, there will be a decline in the body's physiologic function, and the patient will have an incompetent immune status and become at a high risk of complication and respond poorly to ART.

In addition to the above factors, lower level of education, male sex, substance abuse, and unemployment were also mentioned as a significant predictor of mortality by some of the studies included in this review. Another study also reported negative influence of low level of education on mortality among ART users [42]. The more educated patients will have a better understanding of the disease, its treatment, and the instructions given by healthcare providers; as a result, he/she will have enhanced health outcome [43]. The higher rate of mortality in males than females reported in some of the studies reviewed may be because of the males' higher tendency for drug abuse than females in Ethiopia. Earlier health seeking behavior of females might also be a reason for lesser mortality among females than males [44]. This finding was also supported by other studies [11, 25, 26, 28–30, 45].

Even though the inclusion of many homogeneous studies is the strength of this review, there are also some limitations that should be considered in the interpretation of the result of this study. The retrospective nature of the included studies limits the cause and effect relation between death and the different factors reported in the individual studies. Mortality might also be underestimated, since individuals dying at home without being reported were considered as lost to follow-up. On the other hand, HIV-related mortality might also be overestimated since the exact cause of death was not determined and every recorded death in HIV patients was considered as HIV-related.

## 5. Conclusion

5%–40.8% of HIV patients in Ethiopia die in 2–5 years of initiating antiretroviral treatment. Most of the deaths in HIV patients occur early in the course of treatment. The main predictors for death were advanced stage disease, nonworking (bedridden and ambulatory) functional status, lower baseline CD4 count, lower baseline hemoglobin, TB coinfection, lower baseline weight, and poor treatment adherence. Special emphasis and closer follow-up should be given for patients with such characteristics.

## Figures and Tables

**Table 1:** Individual study characteristics.

Sr. number	Author, year of publication	Study area	Study subjects	Study design	Sample size
(1)	Hambisa MT et al., 2013	Nekemte Referral Hospital, East Wollega	Adult HIV patients (age > 14)	Retrospective cohort study	416

(2)	Tsehaineh B. et al., unpublished	JUSH, SW Ethiopia	Adult HIV patients	Retrospective cohort study	832

(3)	Setegn et al., 2015	Goba Hospital, Bale Zone	Adult HIV patients (age >15)	Retrospective cohort study	2036

(4)	Biadgilign S. et al., 2012	Hiwot Fana, Jugal and Dil Chora Hospitals, Eastern Ethiopia	Adult HIV patients	Retrospective cohort study	1537

(5)	Kassa A. et al., 2012	Zewditu Memorial Hospital	Adult HIV patients (aged 15 or more)	Retrospective cohort study	4210

(6)	Seyoum D. et al., 2017	JUSH	Adult HIV patients (age ≥ 18)	Retrospective cohort study	456

(7)	Damtew B. et al., 2015	Kharamara Hospital, Jijiga Town, Eastern Ethiopia	Adult HIV patients (age ≥ 15)	Retrospective cohort study	784

(8)	Alemu AW and Sebastian MS, 2010	Shashemene and Assela Hospitals, Arsi Zone	Adult HIV patients (age ≥ 15)	Retrospective cohort study	272

(9)	Ayalew et al., 2014	Boru Meda and Dessie Referral Hospitals and Kombolcha Health Center	Adult HIV patients (age ≥ 15)	Retrospective cohort study	654

(10)	Ahunie MA and Ebrahim EA, 2017	Debre Tabor General Hospital and Woreta Health Center	Adult HIV patients (age ≥ 15)	Retrospective cohort study	698

(11)	Abebe N et al., 2014	Debremarkos Referral Hospital, NW Ethiopia	Adult HIV patients (age ≥15)	Retrospective cohort study	640

(12)	Tadesse K et al., 2014	Aksum Hospital	Adult HIV patients (age ≥ 15)	Retrospective cohort study	520

(13)	Bedru A. and worku A, unpublished	Zewditu Hospital	Adult HIV patients (age ≥14)	Retrospective cohort study	1070

(14)	Mengesha S et al., 2014	Zewditu Memorial Hospital	Adult HIV patients (age ≥14)	Retrospective cohort study	416

(15)	Moshago T et al., 2012	Mizan Aman Hospital	Adult HIV patients	Retrospective cohort study	2655

(16)	Sapa et al., 2016	Dilla Hospital	Adult HIV patients (age ≥15)	Retrospective cohort study	1391

(17)	Kebebew K and wencheko E, 2012	Armed Forces Teaching and General Hospital	Adult HIV patients (age >15)	Retrospective cohort study	734

**Table 2:** Mortality indicators.

Sr. number	Author, year of publication	Median follow-up period	Death, *n* (%)	Mortality incidence density/100 PY	Mean survival time (95% CI)	Mortality at each period		
						First 3 months	First 6 months	First 12 months
(1)	Hambisa MT et al., 2013	47 months	30 (7.2%)	1.89	NR	NR	17 (56.7%)	21 (70%)

(2)	Tsehaineh B. et al., unpublished	40 months	144 (17.3%)	NR	63.7 months (61.1–66.3)	70 (48.6%)	99 (68.8%)	NR

(3)	Setegn et al., 2015	NR	120 (5.9%)	2.03	34.9 months (33.8–35.9)	NR	NR	78 (65%)

(4)	Biadgilign S. et al., 2012	NR	86 (5.6%)	2.03	NR	NR	NR	63 (73.3%)

(5)	Kassa A. et al., 2012	NR	291 (6.9%)	2.8	NR	NR	166 (57%)	NR

(6)	Seyoum D. et al., 2017	NR	66 (14.5%)	5.3	34 months (22.8–42.0)	NR	NR	40 (60.6%)

(7)	Damtew B. et al., 2015	60 months	87 (11.1)	5.15	20.7 months	49 (56.3%)	NR	NR

(8)	Alemu AW and Sebastian MS, 2010		28 (10.3%)	7	NR	NR	NR	NR

(9)	Ayalew et al., 2014	NR	92 (14.1%)	NR	41.8 months (40.61–43.00)	NR	NR	NR

(10)	Ahunie MA and Ebrahim EA	NR	35 (5.0%)	1.5	NR	NR	NR	NR

(11)	Abebe N et al., 2014	NR	261 (40.8%)	10.74	NR	NR	NR	NR

(12)	Tadesse K et al., 2014	32 months	46 (8.9%)	3.2	NR	NR	NR	27 (59%)

(13)	Bedru A. and worku A, unpublished	34 months	360 (33.6%)	NR	NR	200 (55.6%)	NR	NR

(14)	Mengesha S et al., 2014	34 months	37 (9%)	3.8	39 months	22 (59.5%)	NR	NR

(15)	Moshago T et al., 2012	NR	159 (5.9%)	0.2	89 months	NR	NR	NR

(16)	Sapa et al., 2016	25 months	128 (9.2%)	3.5	NR	33 (26%)	NR	66 (52%)

(17)	Kebebew K and wencheko E, 2012	38.5 months	86 (11.7%)	NR	NR	28 (32.6%)	43 (50%)	86 (100%)

NR: not reported.

**Table 3:** Predictors of mortality.

Sr. number	Author, year of publication	Predictor variable	AHR	95% CI (upper limit, lower limit)
(1)	Hambisa MT et al., 2013	Age ≥ 40	3.055	1.292–7.223
		Baseline hemoglobin level	0.523	0.335–0.816
		Poor ART adherence	27.848	8.928–86.863

(2)	Tsehaineh B. et al., unpublished	Old age	1.03	1.01–1.051
		CD4 count at baseline	0.994	0.992–0.996
		Weight at baseline	0.902	0.816–0.996
		Bedridden functional status	6.904	4.005–11.902
		Ambulatory functional status	2.877	1.899–4.360
		Coinfection with TB	1.906	1.305–2.784
		Substance use	1.42	1.016–1.985

(3)	Setegn et al., 2015	Male	2.67	1.74–4.10
		Bedridden clients	4.4	1.55–12.36
		TB coinfected at ART initiation	4.51	2.86–7.11
		Primary education	0.28	0.11–0.70
		Secondary education	0.34	0.154–0.728
		WHO stage 1	0.16	0.08–0.33
		WHO stage 2	0.34	0.16–0.73
		WHO stage 3	0.24	0.13–0.43

(4)	Biadgilign S. et al., 2012	WHO stage IV	3.19	1.51–6.76
		Bedridden	4.09	2.12–7.90
		>10% weight loss from baseline	4.93	1.20–20.41
		CD4	0.40	0.17–0.93
		Education	2.79	1.26–6.16

(5)	Kassa A. et al., 2012	CD4 < 50 cells/*μ*l	1.80	1.17–2.83
		WHO stage III	1.46	1.03–2.08
		WHO stage IV	2.72	1.91–3.88
		Those who developed TB after ART	1.60	1.19–2.15
		Ambulatory functional status	1.44	1.07–1.93
		Bedridden functional status	2.95	2.10–4.13

(6)	Seyoum D. et al., 2017	Age > 35	3.8	1.6–9.1
		Baseline weight	0.93	0.90–0.97
		Baseline WHO stage IV	6.2	2.2–14.2
		Low adherence to ART	4.2	2.5–7.1

(7)	Damtew B. et al., 2015	Single marital status	2.31	1.18–4.50
		Bedridden functional status	5.91	2.87–12.16
		Advanced WHO stage	7.36	3.17–17.12
		BMI < 18.5 kg/m^2^	2.20	1.18–4.09
		CD4 count < 50 cells/*μ*L	2.70	1.26–5.80
		Severe anemia	4.57	2.30–9.10
		TB coinfection	2.30	1.28–4.11

(8)	Alemu AW and Sebastian MS, 2010	Hemoglobin < 10 g/dL	2.56	1.11–5.88
		WHO stage IV	5.13	2.33–11.33
		Not on cotrimoxazole prophylaxis	7.14	2.7–20.00

(9)	Ayalew et al., 2014	Age 30–40	0.49	0.28–0.85
		Rural residency	1.74	1.11–2.74
		CD4 count	0.998	0.996–0.999
		Weight	0.968	0.943–0.993
		Not working functional status	3.62	1.96–6.68
		Lymphocyte count	0.969	0.945–0.994
		WHO stage IV	2.38	1.21–4.63
		TB positive	1.87	1.03–3.40

(10)	Ahunie MA and Ebrahim EA	Ambulatory functional status	4.2	1.7–10.7
		Bedridden functional status	6.5	2.0–20.7
		Poor antiretroviral drug adherence	5.1	1.6–16.3

(11)	Abebe N et al., 2014	Baseline hemoglobin < 10 g/mm^3^	1.86	1.39–2.64
		Ambulatory functional status	2.72	1.90–3.90
		Bedridden functional status	2.38	1.32–4.27
		WHO stages III and IV	2.16	1.10–4.25
		Poor adherence	2.16	1.03–4.56
		Fair adherence	1.88	1.08–3.29
		Unexplained chronic diarrhea	1.53	1.09–2.15
		Not on TB prophylaxis	3.98	1.87–8.44

(12)	Tadesse K et al., 2014	Hemoglobin level < 11 mg/dl	1.9	1.01–3.52
		CD4 cell count < 50 cells/*μ*l	2.1	1.13–3.89
		Male gender	1.9	1.01–3.52
		Weight < 40 kg	2.3	1.24–4.55
		Primary and lower level of education	2.6	1.29–5.55

(13)	Bedru A. and worku A, unpublished	Poor ART adherence	3.92	3.13–4.90
		Advanced WHO staging	2.47	1.58–3.81
		Being unemployed	1.87	1.49–2.34
		Moderate anemia	1.86	1.35–2.56
		Low CD4 count	1.85	1.35–2.52

(14)	Mengesha S et al., 2014	WHO clinical stage	2.99	1.26–5.31
		Anemia	5.54	2.58–11.86
		Having past TB coinfection	4.13	1.79–9.51

(15)	Moshago T et al., 2012	WHO clinical stage IV	4.5	1.36–14.88
		WHO clinical stage III	3.2	1.06–10.24
		History of TB coinfection	1.25	1.03–1.53
		Bedridden functional status	2.63	2.05–3.37
		Ambulatory functional status	1.56	1.31–1.86

(16)	Sapa et al., 2016	BMI < 18.5 kg/m^2^	3.12	1.39–7.76
		CD4 cell count < 50 cells/mm^3^	4.55	1.19–8.44
		Drug addiction	2.03	1.11–4.56
		WHO stages III and IV	11.25	8.67–17.96
		Severe anemia	5.14	3.12–9.65

(17)	Kebebew K and Wencheko E, 2012	CD4 cell count at baseline	0.78	0.64–0.95
		Employment status	2.31	1.25–4.28
		Ambulatory functional status	2.01	1.02–3.98
		Bedridden functional status	3.36	1.73–6.50
		WHO clinical stage III	7.05	1.68–29.66
		WHO clinical stage IV	12.64	3.00–53.20
		TB coinfection	1.73	1.04–2.89
		Presence of opportunistic infections	8.99	1.24–65.09

## Abbreviations

AIDS: Acquired immune deficiency syndrome
ART: Antiretroviral therapy
BMI: Body mass index
HAART: Highly active antiretroviral therapy
HIV: Human immunodeficiency virus
PY: Person-years
TB: Tuberculosis.
